# Knowledge, attitudes and prevention practices related to dog-mediated rabies in Ethiopia: a systematic review and meta-analysis of observational epidemiological studies from inception to 2023

**DOI:** 10.3389/fpubh.2023.1276859

**Published:** 2023-12-21

**Authors:** Beshada Zerfu Woldegeorgis, Amanuel Paulos Genebo, Amanuel Yosef Gebrekidan, Gizachew Ambaw Kassie, Gedion Asnake Azeze, Yordanos Sisay Asgedom

**Affiliations:** ^1^Department of Internal Medicine, College of Health Sciences and Medicine, Wolaita Sodo University, Wolaita Sodo, Ethiopia; ^2^School of Public Health, College of Health Sciences and Medicine, Wolaita Sodo University, Wolaita Sodo, Ethiopia; ^3^Department of Midwifery, College of Medicine and Health Sciences, Hawassa University, Hawassa, Ethiopia; ^4^Department of Epidemiology, College of Health Sciences and Medicine, Wolaita Sodo University, Wolaita Sodo, Ethiopia

**Keywords:** rabies, attitudes, practices, knowledge, Ethiopia, systematic review

## Abstract

**Background:**

Rabies is a horrific and neglected zoonotic disease that kills thousands of people worldwide each year and continues to pose threats to public health. Prevention and control of dog-transmitted rabies require mapping the level of understanding, perception, and existing practices to minimize its impacts on health. Therefore, we undertook this systematic review and meta-analysis to pool evidence from available data on knowledge, attitudes, and prevention practices regarding the disease from studies conducted in various areas of Ethiopia.

**Methods:**

Articles were searched in electronic bibliographic medical databases such as the Excerpta Medica database, PubMed, Web of Science, African Journals Online, Google Scholar, and Scopus. We used Microsoft Excel spreadsheets and STATA software version 16 for the data excerption and analysis, respectively. The variability among studies was evaluated via Higgins and Thompson’s *I*^2^ statistics and the *x*^2^ test (significant at *p* ≤ 0.1). The Dersimonian and Laird random-effect meta-analysis model was used to estimate the pooled effect at a 95% uncertainty interval (UI). Visual inspection and Egger’s test (significant at *p* ≤ 0.05) were used to identify the presence of small-study effects.

**Results:**

The search identified 1,249 electronic records. Of them, 27 studies involving 11,150 participants met the inclusion criteria. The pooled prevalence of a good level of knowledge was 62.24% (95% UI: 48.56, 75.92). Furthermore, the pooled prevalence of a favorable level of attitudes towards rabies and a good level of rabies prevention practices was only 56.73% (95% UI: 47.16, 66.29) and 52.73% (95% UI: 43.32, 62.15), respectively.

**Conclusion:**

The study revealed credible gaps in attitudes and prevention practices, though some level of knowledge about dog-mediated rabies was demonstrated. Therefore, we call for country-wide cross-sectoral collaboration to allow for the realization of a global elimination strategy for dog-mediated human rabies.

## Introduction

Rabies, an acute lethal infectious disease of the central nervous system in humans and animals that is caused by the rabies virus from the family *Rhabdoviridae*, genus *Lyssavirus*, remains one of the foremost neglected viral zoonotic diseases of the 21st century (1). Rabies is a vaccine-preventable disease readily transmitted through the bite of an infected animal and is a nearly uniformly fatal disease (1, 2). Dogs are the most important rabies reservoirs, accounting for more than 99% of human rabies cases (3). Globally, an estimated 59,000 people die each year from dog-mediated human rabies, with Africa (36.4%) and Asia (59.6%) having the highest *per capita* fatality rates (4). Rural people and children under the age of 15 (40%) are disproportionately affected, particularly in resource-limited areas or resource-poor nations. Furthermore, the World Health Organization (WHO) estimates that rabies costs the world approximately 8.6 billion US dollars each year (2).

In Ethiopia, a retrospective review of human rabies exposure cases in Addis Ababa sub-cities (2015–2019) revealed a cumulative incidence ranging from 0.1 to 24.8 per 100,000 inhabitants per year (5), and in the Tigray region (2012–2015), human rabies exposure cases ranging from 35.8 to 89.8 per 100,000 inhabitants per year (6) were reported, although the actual estimate of the problem is expected to be higher because the community’s health-seeking practice for rabies is limited (7). Ethiopia ranked second among African countries in terms of dog-mediated rabies mortality (8). More importantly, estimates suggest that human rabies was responsible for an estimated 3,000 deaths each year, resulting in 194,000 disability-adjusted life years and 2 million US dollars in treatment costs per year for 97,000 potentially exposed people in Ethiopia (9).

The 2030 WHO’s global strategic plan to achieve zero dog-mediated rabies deaths in humans (10) would contribute towards attaining the United Nations’ sustainable development goal of ending epidemics of neglected tropical diseases (NTD), malaria, tuberculosis, and acquired immunodeficiency deficiency syndrome (11). It requires engaging communities, healthcare workers, and other stakeholders to build awareness of rabies, and dog vaccination, particularly in settings with a high incidence of dog bites and unrestrained dogs (12). Nevertheless, according to Lankau et al. (13) significant rabies knowledge gaps were found in South Carolina, a state in the United States. In addition, a One Health approach survey of rabies knowledge gaps among human and animal healthcare practitioners in Washington found huge knowledge gaps among veterinarians and physicians (14), and in Vietnam, where the cumulative incidence of rabies ranged from 1.7 to 117.2 per 100,000 population (15), one in ten public health workers did not know that the rabies virus could be transmitted by infected animals (16).

In Africa, a Senegalese research report found that only 35.8%, 26.3%, and 45.3% of healthcare professionals had adequate knowledge, favorable attitudes, and good practices, respectively (17). Furthermore, the majority of dog owners in Rwanda’s Kigali City had insufficient knowledge about rabies (only 43.1% knew that rabies causes fatal illness, and only 20.4% had adequate knowledge about cleaning dog bite wounds) (18). Poor perceptions about rabies control and prevention are a major issue that hinders efforts to bring dog-mediated rabies deaths to zero (19).

In Ethiopia, human rabies is locally called “*Yebed wusha beshita*”, “*Kelebat*”, and “*Likefit*”, all meaning “a mad dog disease” (20). Bodies of evidence suggest Ethiopia is still in the early stages of rabies control (8). In addition, the burden of human rabies is high, with significant variation across areas in Ethiopia (9); however, nationally representative (baseline) research into the knowledge, attitudes, and practices (KAP) has been lacking, although there are individual reports with discrepant results from various areas in the country. Keeping with this, we aimed to do the following: (1) estimate the pooled prevalence of a good level of knowledge, (2) estimate the pooled prevalence of favorable attitudes, (3) estimate the pooled prevalence of a good level of rabies prevention practices, and (4) identify the factors associated with a good level of KAP towards dog-mediated rabies.

## Methods

### Study protocol registration and reporting

Before commencing data extraction, we submitted a full study protocol, written based on the Preferred reporting items for systematic review and meta-analysis protocols (PRISMA-P) 2015 (21), to an international database, the Prospective Register of Systematic Reviews (PROSPERO) for registration. To that end, the guidance notes for registering systematic reviews of human studies, which include the following activities, were strictly followed: (1) checking the inclusion criteria to make sure that the review is eligible for inclusion in PROSPERO and avoid wasting time, (2) ensuring that the review protocol is in its (near) final form and that no significant changes are anticipated at this stage, (3) searching PROSPERO to ensure that another member of the team has not already registered the review, and (4) checking the PROSPERO to see if a similar study has already been done to avoid duplicating a study that is being done by other researchers or has been registered previously. The study protocol was registered^1^ with registration number CRD42023437439 following the online submission of the completed records. The protocol has been amended since registration with a minor change to the title, and the overall review progress was updated after the completion of the data analysis. To effect this, revision notes were entered by providing a brief description of the minor changes made. We reported the systematic review and meta-analysis results using the Preferred Reporting Items for Systematic Reviews and Meta-Analyses (PRISMA) 2020 checklist (22) (Supplementary File S1).

### Eligibility criteria

Studies were recruited according to the criteria outlined in the methodological guidance for conducting systematic reviews of prevalence and incidence data, which recommends strict adherence to the CoCoPop mnemonic (condition, context, and population) (23).

Population/type of participants: We included studies involving persons from the community, healthcare workers, animal health practitioners or veterinarians, dog bite victims, or persons receiving anti-rabies prophylaxis at health facilities.

Condition/domain: Studies that clearly stated and defined the outcome of interest based on an individual’s knowledge, attitudes, or practices toward human rabies were included. Context/settings: Observational epidemiological studies (cross-sectional, case–control, follow-up) were considered. Studies conducted in Ethiopia and reports in the English language from inception to June 18, 2023, and published in international and domestic peer-reviewed journals were included. Studies without full-text access; articles that did not contain required information on the outcomes of interest; studies published in non-open access journals; findings from personal opinions; articles reporting outside the scope of the outcome of interest; qualitative study design; case reports; case series; letters to editors; and unpublished data were excluded.

### Information sources and search strategy

The search was carried out in the following electronic bibliographic medical databases: Excerpta Medica database, PubMed, Web of Science, African Journals Online, Google Scholar, and Scopus, to ensure complete coverage of the topic by accounting for variability between the indexing in each database. The reference lists of final articles included in the quantitative synthesis were scanned to ensure literature saturation. Where necessary, we also searched the authors’ files to ensure that all relevant materials had been captured. Literature search strategies were developed using medical subject headings (MeSH) and text words related to the outcomes of interest. For the advanced search in PubMed, the following steps comprised the search process: initially, the search terms were developed along four domains: “rabies,” “knowledge,” “attitude,” and “practices.” As such, we gathered keywords from Google Scholar, Wikipedia, and Google for each concept, which was then searched independently in PubMed to find MeSH terms in the MeSH hierarchy tree and then combined in an advanced search. Boolean logic (“AND” and “OR”) was used to combine these concepts. The database search was double-blinded and conducted from May 1, 2023, to June 18, 2023, by two authors (BW and YA). The search terms, with their Boolean operators, are supplied as an additional file (Supplementary File S2).

### Study selection procedures

The articles found through the electronic database searches were exported to the reference management software, EndNote X7, where duplicate studies were then eliminated. Two authors (BW and YA) independently screened the titles and abstracts that were obtained by the search against the inclusion criteria. To describe the extent to which the assessments by both authors were similar, inter-rater agreement was calculated after referring to the Cochrane Handbook for systematic reviews (24). Thus, a value of kappa 0.75 or more was considered, reflecting excellent agreement. The screened articles were then subjected to a full article review by two independent authors (APG and GK). Pre-specified criteria for inclusion in the review were followed to determine which records were relevant and should be included. Where more information was required to answer queries regarding eligibility, the remaining authors were involved. Disagreements about whether a study should be included were resolved by discussion. Moreover, the reasons for excluding the articles were recorded at each step.

### Data extraction

Two authors (GA and GK), working independently, excerpted the relevant data from the studies using a standardized Microsoft Excel spreadsheet. For data extraction, Joana Briggs Institute (JBI) data collection formats suitable for meta-analysis were employed (25). The data extraction format captured data on the following main components: information about data extraction from reports (name of data extractors, date of data extraction, and study identification number), study authors, year of publication of the article, study methods (study design, statistical analysis), study settings (regions, and specific areas from which study participants recruited), population characteristics (sex, age), information related to the pre-specified outcome domain in this systematic review (i.e., KAP related to human rabies), measurement tool or instrument (including the definition of a threshold for a good level of knowledge, a favorable level of attitudes, and a good level of practices toward rabies), and information related to the resultsfor each study included in the quantitative analysis (number of participants included in the analysis, and the non-response rate). The reliability agreement among the data extractors was evaluated and verified using Cohan’s kappa coefficient after data was recovered from 30% of the primary studies (26). The kappa coefficient’s strength of agreement was divided into five categories: low (0.20), fair (0.21–0.40), moderate (0.41–0.60), good (0.61–0.80), and virtually perfect agreement (0.81–1). A kappa statistic value of more than or equal to 0.5 was regarded as congruent and acceptable. In the case of disagreements between the two data extractors, a third author (YA) was involved in adjudicating unresolved disagreements through discussion and re-checking of the original articles.

### Outcome measurement

The primary outcome of interest was the proportion of the participants who had a good level of knowledge, favorable attitudes, and good practices toward rabies. Bloom’s proposed cut-off point above 60% was adopted to classify the pooled estimates into good knowledge, favorable attitude, and good practice (27). The secondary outcome was factors associated with KAP towards rabies. We found only four studies (28–31) that reported associated factors. As such, due to the lack of information to carry out a meta-regression of effect measure, we presented associated factors in a summary of findings.

### Risk of bias (quality) assessment

JBI’s critical appraisal tools for descriptive, and analytic cross-sectional study design were used (32). As a result, 24 articles (20, 28, 33–54) were evaluated by two authors (AYG and GK) against the following constructs: (1) appropriateness of the sample frame to address the target population, (2) appropriateness of participant sampling, (3) study size adequacy, (4) whether the study subjects and the setting were described in detail, (5) whether valid methods were employed to identify the domain, (6) whether the domain was measured in a reliable and standard way for all participants, (7) the presence of appropriate statistical analysis, and (8) whether the response rate was adequate and if not, how it was managed (Supplementary File S3). The remaining three primary articles (29–31) were evaluated by an eight-construct JBI critical checklist tool designed for analytical cross-sectional epidemiological study design. Thus, the main components included the following: (1) whether the eligibility criteria were defined, (2) whether study settings and subjects were described in detail, (3) whether the exposure was measured accurately, (4) whether the measurements used were standard and objective, (5) whether the confounding factors were identified, (6) whether addressing the confounding factor was described in detail, (7) whether the outcome was measured accurately and (8) whether statistical methods were appropriate (Supplementary File S4). The total score was determined by counting the “yes” responses to each question and adding them. Articles with appraisal scores of seven or more were deemed suitable to be included in the quantitative analysis. When disagreements arose, they were settled by consulting with a third independent author (GA).

### Data synthesis

Extracted data were imported from Microsoft Excel 2010 into Stata 16 MP version for analysis. The presence and extent of variability among studies (inconsistency or heterogeneity) were evaluated graphically (present when the uncertainty interval for the results of individual studies generally depicted in forest plots using the horizontal lines have poor overlap) and more formally, using statistical methods (the Cochrane chi-squared test, included in the forest plots, the threshold for statistical significance was set at *p* ≤ 0.1; Higgins and Thompson’s *I*^2^ statistics: 0% to 40%: may not be important; 30% to 60% may represent moderate heterogeneity; 50% to 90%: may represent substantial heterogeneity; 75% to 100%: considerable heterogeneity) (24). We employed the random-effect meta-analysis model (REM) to estimate Der Simonian and Laird’s pooled effect, as considerable statistical heterogeneity was observed (Higgins and Thompson’s I^2^ statistics was ≥50% and *p* value was ≤0.1). Moreover, subgroup analyses (based on sample size population, and study region as covariates), meta-regression (based on year of publication, and sample size as covariates), and sensitivity analyses were performed to explore the possible sources of heterogeneity. To evaluate the presence of small study effects, publication bias was explored through statistical methods (Egger test: significant at *p* ≤ 0.05) and graphical approaches (funnel plots) (55). The symmetrical distribution of the points about the summary effect size is an indication of the absence of a possible small-study effect or publication bias. However, any asymmetrical distribution of the points (the typical pattern in the presence of small-study effects is a prominent asymmetry at the bottom that progressively disappears as we move up to larger studies) may support the presence of a possible small-study effect or publication bias (55, 56).

## Results

### Search and study selection

**Figure 1:**
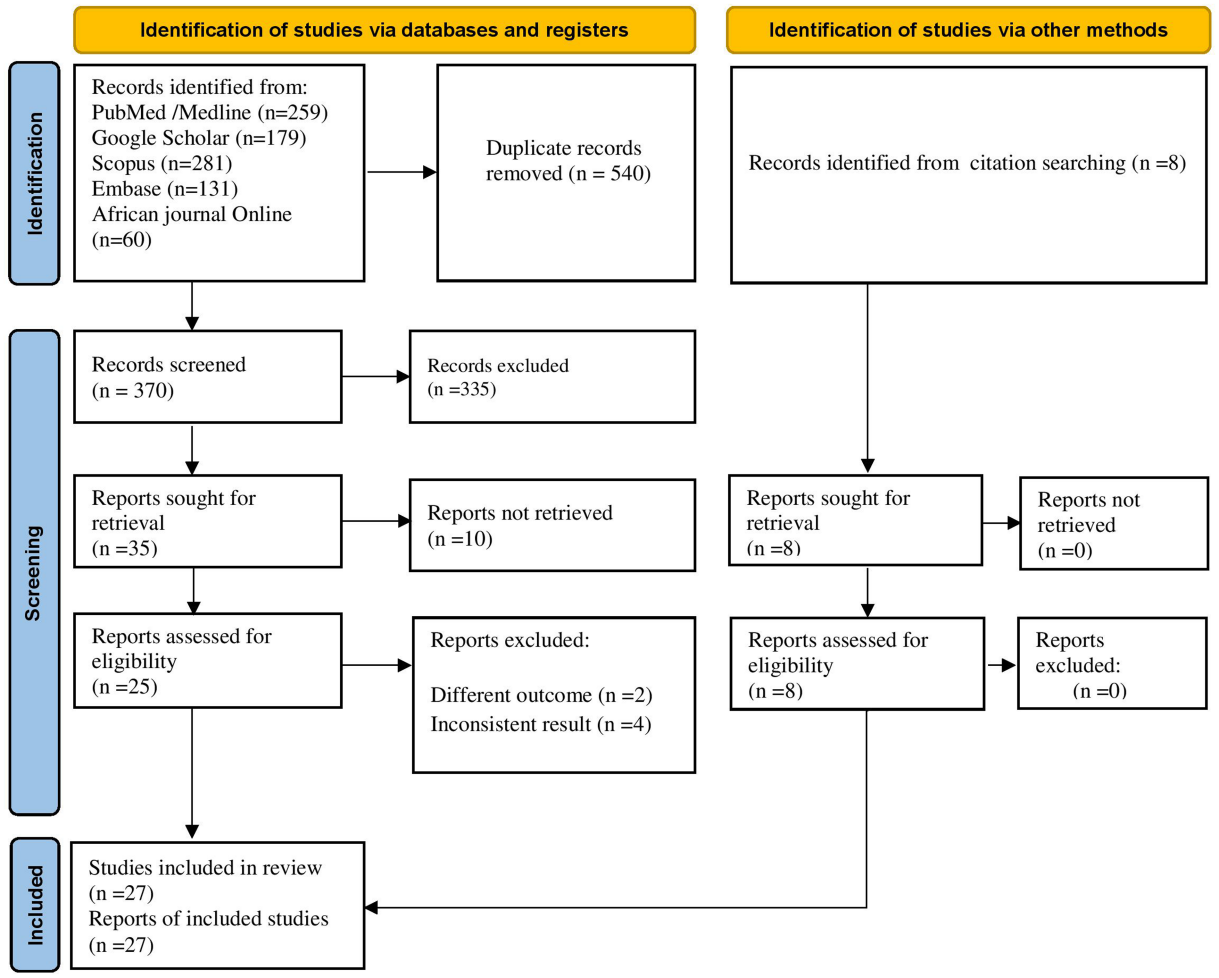
PRISMA flow diagram for identification and selection of articles included in this review.

Through database searching, 910 articles were identified. Due to duplication, 540 articles were removed. The remaining 370 were screened based on their title and abstracts, with 335 being removed as unrelated to our domain. Finally, 25 full-text primary articles were evaluated against eligibility criteria and 6 of them were removed (inconsistent results, *n* = 4, and different outcome, *n* = 2). In addition, through citation searching, eight articles were retrieved. Finally, 27 articles were selected for quantitative analysis (Figure 1).

### Study characteristics

The systematic review and meta-analysis included a total of 27 eligible studies, with 11,150 participants (20, 28–31, 33–54). The study’s sample size ranged from 120 (45) to 1,240 (53) subjects. The response rate was between 90.9% (39) and 100% (28–31, 33–37, 40–54). About 60.41% (*n* = 6,736) of the participants were male. Twenty-six of the epidemiological studies were cross-sectional (20, 28–31, 33–44, 46–54), and one was a prospective follow-up study (45). All studies, except one (49), employed probability sampling techniques. Furthermore, an interviewer-administered questionnaire (20, 28–31, 33–43, 45–54) was used in all, and a study used both an interviewer-administered questionnaire and a review of secondary data (44). Except for a study by Kabeta et al. (49) that was carried out among dog bite victims attending Jimma Town anti-rabies Health Center located in the Oromia region, the remaining 26 studies were conducted in the community (20, 28–31, 33–48, 50–54). Twelve of the studies were published in the previous five years (2018–2023 Gregorian Calendar) (28, 30, 31, 34–36, 38, 40, 42, 47, 48, 52). Eleven of the studies were conducted in the Amhara region (20, 28, 31, 35, 39, 41, 44, 45, 47, 51, 52), 9 in the Oromia region (29, 30, 33, 38, 42, 46, 49, 50); 3 in Addis Ababa the capital of Ethiopia’s (37, 43, 53); 2 in the Southern Nations, Nationalities, and Peoples’ Region (SNNPR) (34, 54), 1 each in Tigray (40), and Somalia (36) regions. The individual study estimates range from 7.50% (39) to 99.25% (51) for a good level of knowledge about rabies, 12.55% (53) to 95.57% (49) for a favorable level of attitudes, and 4.69% (52) to 85.68% (28) for a good level of practices towards rabies prevention (Table 1).

**Table 1:** The characteristics of the studies included in the systematic review and meta-analysis.

SN	Authors	Year	Region/ chartered city	Study design	Population	Study size	Good knowledge, *n* (%)	Favorable attitudes, *n* (%)	Good practices, *n* (%)	Response rate (%)	Score
1	Alie et al. (39)	2015	Amhara	Cross-sectional	Community	384	190 (49.48)	190 (49.48)	190 (49.48)	90.9	8
2	Hagos et al. (40)	2020	Tigray	Cross-sectional	Community	633	354 (55.92)	354 (55.92)	388 (61.30)	100	8
3	Guadu et al. (20)	2014	Amhara	Cross-sectional	Community	410	263 (64.14)	229 (55.85)	249 (60.73)	96.9	8
4	Yalemebrat et al. (41)	2016	Amhara	Cross-sectional	Community	416	251 (60.33)	251 (60.33)	251 (60.33)	100	8
5	Gebremeskel et al. (28)	2019	Amhara	Cross-sectional	Community	384	329 (85.68)	329 (85.68)	329 (85.68)	100	9
6	Jemberu et al. (45)	2013	Amhara	Cross-sectional	Community	120	118 (98.33)	Not reported	Not reported	100	9
7	Bahiru et al. (47)	2022	Amhara	Follow up	Community	889	549 (61.75)	648 (72.90)	402 (45.22)	100	8
8	Kabeta et al. (49)	2015	Oromia	Cross-sectional	Bite victims	384	352 (91.67)	367 (95.57)	165 (42.97)	100	8
9	Abdela et al. (50)	2017	Oromia	Cross-sectional	Community	135	71 (52.59)	70 (51.85)	70 (57.85)	100	8
10	Digafe et al. (51)	2015	Amhara	Cross-sectional	Community	400	397 (99.25)	271 (67.75)	190 (47.50)	100	8
11	Ahmed et al. (30)	2022	Oromia	Cross-sectional	Community	326	170 (52.15)	160 (52.15)	154 (47.24)	100	8
12	Wolelaw et al. (31)	2022	Amhara	Cross-sectional	Community	609	349 (57.31)	312 (52.23)	264 (43.35)	100	8
13	Bihon et al. (52)	2020	Amhara	Cross-sectional	Community	384	188 (48.96)	188 (48.96)	188 (4.69)	100	8
14	Ali et al. (53)	2013	Addis Ababa	Cross-sectional	Community	1,240	93 (7.50)	230 (12.55)	143 (11.53)	100	8
15	Mamuye et al. (54)	2016	SNNPR	Cross-sectional	Community	410	220 (53.66)	220 (53.66)	220 (53.66)	100	8
16	Gebeyaw et al. (35)	2020	Amhara	Cross-sectional	Community	138	23 (16.67)	40 (28.99)	38 (27.54)	100	8
17	Newayeselassie et al. (37)	2012	Addis Ababa	Cross-sectional	Community	315	286 (90.80)	192 (60.96)	Not reported	100	8
18	Tolosa and Mengistu (46)	2017	Oromia	Cross-sectional	Community	384	204 (53.13)	204 (53.13)	204 (53.13)	100	8
19	Abdela and Teshome (29)	2017	Oromia	Cross-sectional	Community	150	86 (57.33)	86 (57.33)	86 (57.33)	100	8
20	Birasa et al. (38)	2020	Oromia	Cross-sectional	Community	400	352 (88.00)	108 (27.00)	108 (27.00)	94.8	8
21	Gumi et al. (42)	2019	Oromia	Cross-sectional	Community	162	94 (58.02)	78 (48.15)	79 (48.77)	100	8
22	Abera et al. (43)	2012	Addis Ababa	Cross-sectional	Community	384	258 (67.12)	258 (67.12)	258 (67.19)	100	8
23	Dabuma et al. (33)	2017	Oromia	Cross-sectional	Community	386	178 (46.11)	195 (50.52)	245 (63.47)	100	8
24	Jama and mengistu (36)	2023	Somali	Cross-sectional	Community	384	274 (71.35)	274 (73.35)	274 (71.35)	100	8
25	Fesseha and Abebe (34)	2020	SNNPR	Cross-sectional	Community	330	282 (85.45)	282 (85.45)	282 (85.45)	100	8
26	Tamiru et al. (48)	2022	Oromia	Cross-sectional	Community	633	354 (55.92)	354 (55.92)	354 (55.92)	100	8
27	Wassihune et al. (44)	2017	Amhara	Cross-sectional	Community	360	184 (51.11)	184 (51.11)	184 (51.11)	100	8

SNNPR, the Southern Nations, Nationalities, and Peoples’ Region.

### Knowledge, attitudes, and prevention practices

**Figure 2:**
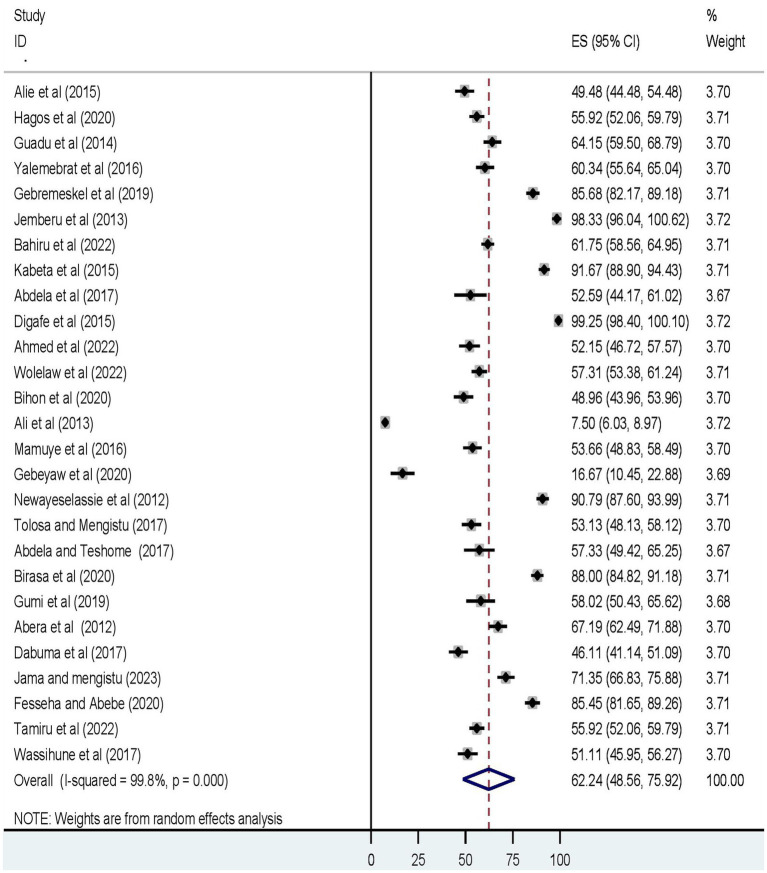
The forest plot displays the meta-analysis result on the prevalence of a good level of knowledge about dog-mediated rabies under the random-effects model (overall).

Twenty-seven original studies conducted in various settings in Ethiopia were deemed eligible and included in the quantitative analysis. The REM revealed that the pooled prevalence of a good level of knowledge was 62.24% (95% UI: 48.56, 75.92) (Figure 2).

**Figure 3:**
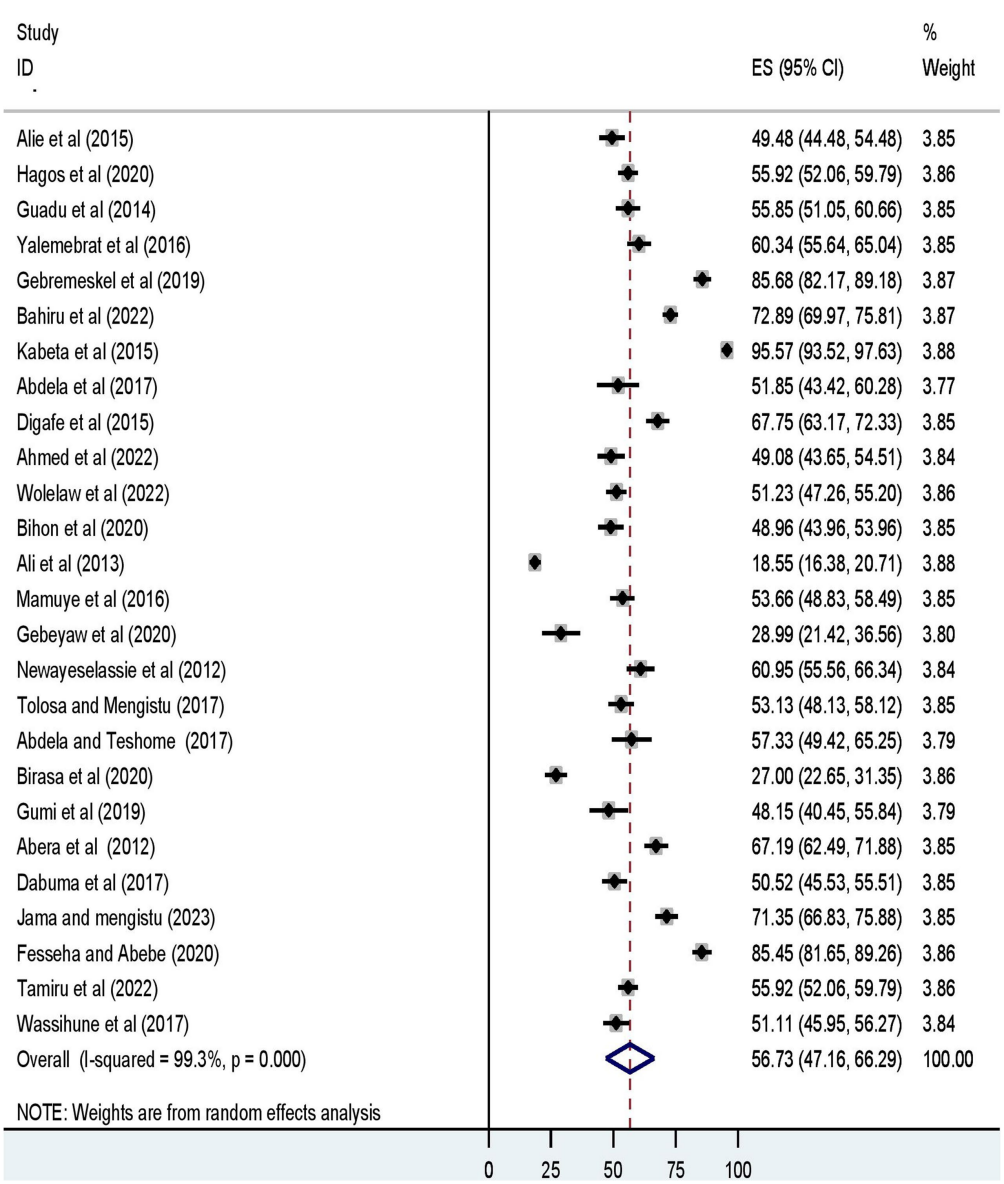
The forest plot displays the meta-analysis result on the prevalence of a favorable level of attitude towards dog-mediated rabies under the random-effects model (overall).

**Figure 4:**
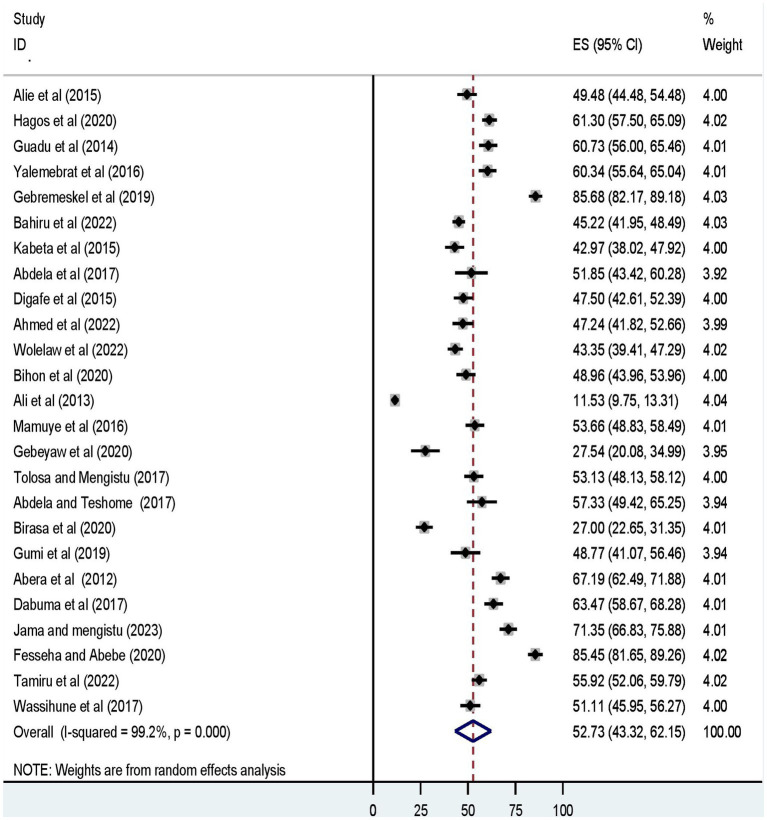
The forest plot displays the meta-analysis result on the prevalence of a good level of dog-mediated rabies prevention practices under the random-effects model (overall).

Furthermore, the pooled prevalence of a favorable level of attitudes towards rabies, and a good level of rabies prevention practices was 56.73% (95% UI: 47.16, 66.29) (Figure 3), and 52.73% (95% UI: 43.32, 62.15) (Figure 4), respectively.

### Heterogeneity

#### Subgroup (subset) meta-analysis

To identify the source of statistical heterogeneity, we undertook a subgroup random-effect meta-analysis for subsets of study regions, sample size partitioned into <385 and ≥ 385, and population split into community members and dog bite victims. This study found that 91.67% (95% UI 88.90, 94.43), and 95.57% (95% UI 93.52, 97.63) of dog bite victims had a good level of knowledge and favorable attitudes toward rabies, respectively. On the other hand, a good level of rabies prevention practices was 69.59% (95% UI 38.43, 100.75) in the SNNPR (Table 2).

**Table 2:** Results of subgroup meta-analysis of good knowledge, favorable attitudes, and good prevention practices towards dog-mediated rabies in Ethiopia.

Domain	Characteristics	No. of studies	Subtotal estimate (95% UI)	Heterogeneity	
				*I*^2^ (%)	*p* value
Good knowledge	Study size				
	<385	16	64.45 (53.80, 75.20)	99.0	<0.001
	≥385	11	59.09 (32.40, 85.77)	99.9	<0.001
	Population				
	Community	26	61.11 (46.84, 75.38)	99.8	<0.001
	Dog bite victims	1	91.67 (88.90, 94.43)		
	Region				
	Amhara	11	63.10 (48.30, 77.89)	99.6	<0.001
	Oromia	9	61.77 (48.29, 75.25)	98.7	<0.001
	Addis Ababa	3	55.14 (−4.42, 114.70)	99.9	<0.001
	SNNPR	2	62.24 (48.56, 75.92)	99.0	<0.001
	Others	2	63.59 (48.47, 78.71)	96.1	<0.001
Favorable attitudes	Study size				
	<385	15	60.41 (49.58, 71.25)	99.8	<0.001
	≥385	11	51.77 (39.10, 64.44)	99.2	<0.001
	Population				
	Community	25	55.17 (46.93, 63.40)	98.9	<0.001
	Dog bite victims	1	95.57 (93.52, 97.63)		
	Region				
	Amhara	8	59.22 (48.47, 69.98)	97.9	<0.001
	Oromia	11	53.55 (37.81, 69.29)	99.3	<0.001
	Addis Ababa	3	48.84 (13.53, 84.15)	99.6	<0.001
	SNNPR	2	69.59 (38.43, 100.75)	99.0	<0.001
	Others	2	63.59 (48.47, 78.71)	99.3	<0.001
Poor prevention practices	Study size				
	<385	14	56.41 (47.01, 65.81)	97.9	<0.001
	≥385	11	48.15 (34.63, 61.68)	99.3	<0.001
	Population				
	Community	24	53.14 (43.37, 62.91)	99.2	<0.001
	Dog bite victims	1	42.97 (38.02, 47.92)		
	Region				
	Amhara	10	52.09 (42.08, 62.11)	98.0	<0.001
	Oromia	9	49.67 (41.69, 57.65)	95.0	<0.001
	Addis Ababa	2	39.32 (−15.23, 93.86)	99.8	<0.001
	SNNPR	2	69.59 (38.43, 100.75)	99.0	<0.001
	Others	2	66.23 (56.39, 76.10)	91.0	0.001

SNNPR, the Southern Nations, Nationalities, and Peoples’ Region; UI, uncertainty interval.

#### Meta-regression

We further performed meta-regression analyses to explore the cause of heterogeneity, using the sample size and year of publication as covariates at 5% statistical significance for a good level of knowledge, a favorable level of attitudes, and a good level of practices towards rabies. As shown in Table 3, these covariates were not found to be the cause of statistical heterogeneity.

**Table 3:** Meta-regression analysis of factors affecting study heterogeneity.

Covariates	Coefficient	Standard error	*t*	*p* > |*t*|	95% UI	
Good knowledge						
Sample	−0.0320742	0.0176784	−1.81	0.082	−0.0685607	0.0044122
Year of publication	−0.7515344	1.262297	−0.60	0.557	−3.356787	1.853718
Favorable attitudes						
Sample	0136742	0.0151838	−0.90	0.377	−0.0450844	0.0177359
Year of publication	0.1100006	1.093434	0.10	0.921	−2.15194	2.371941
Good prevention practices						
Sample	−0.0251814	0.0136035	−1.85	0.078	−0.0533933	0.0030306
Year of publication	0.5223931	1.046438	0.50	0.623	−1.647786	2.692572

#### Sensitivity meta-analysis

**Figure 5:**
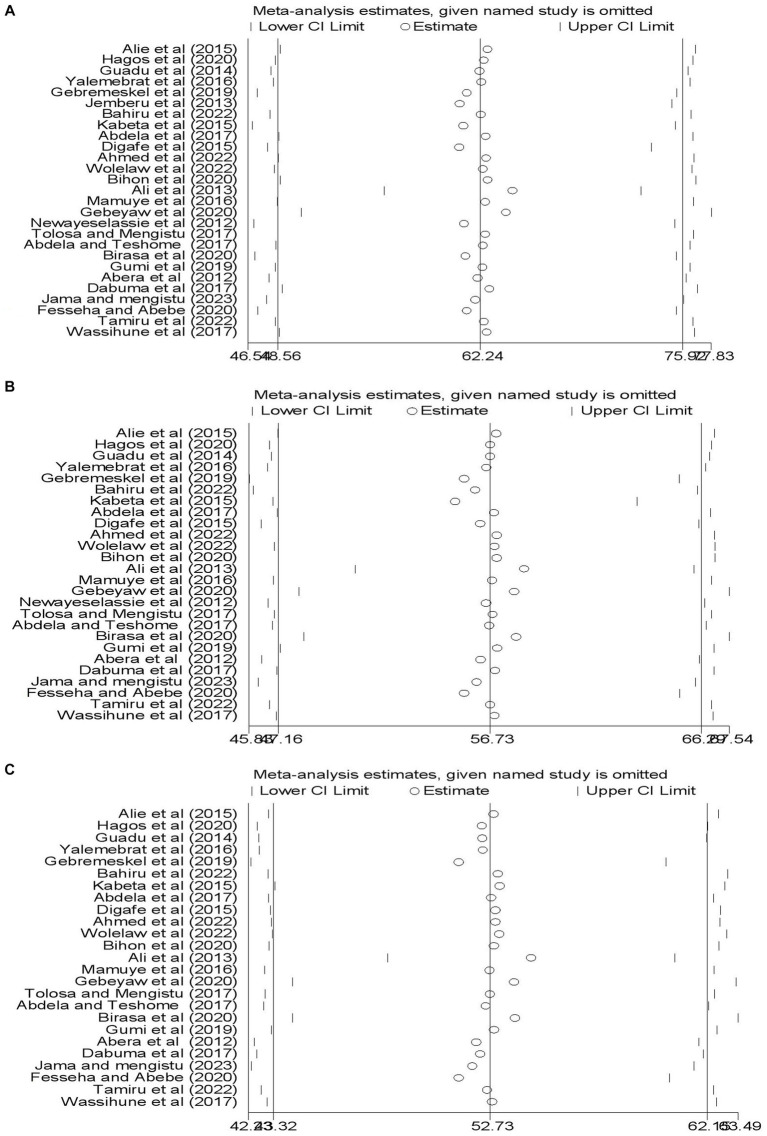
Illustration of sensitivity meta-analysis. **(A)** A good level of knowledge. **(B)** A favorable attitudes. **(C)** A good prevention practices related dog mediated human rabies.

A leave-out-one sensitivity analysis was conducted to assess the impact of each study on the pooled level of good knowledge, favorable attitudes, and good practices toward rabies while gradually excluding each study. Results showed that the combined effects did not significantly change as a result of the excluded study (Figures 5A–C).

#### Publication bias

**Figure 6:**
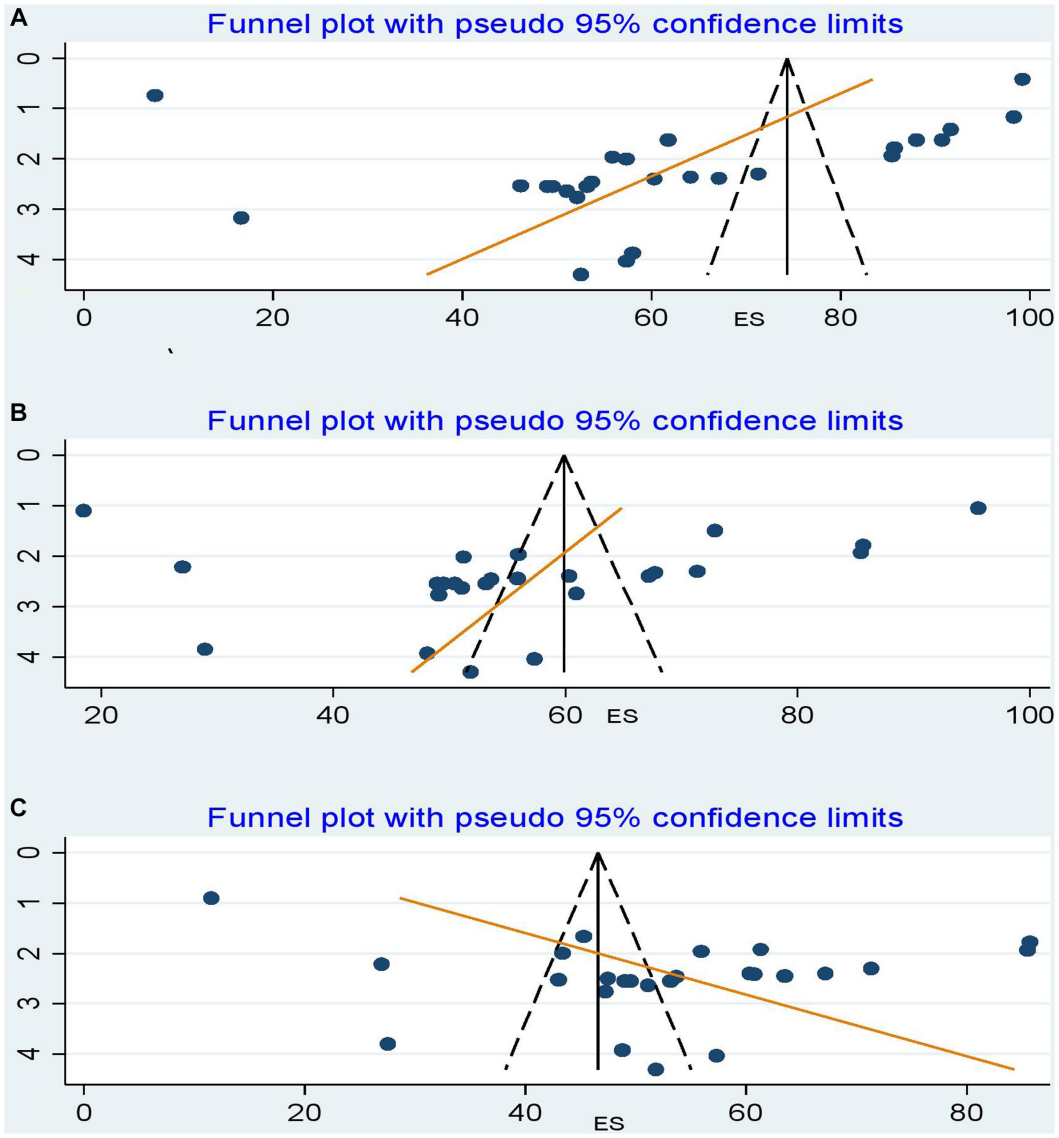
Funnel plots of publication biases. **(A)** A good level of knowledge about dog-mediated human rabies. **(B)** A favorable level of attitudes towards dog-mediated human rabies. **(C)** A good level of dog-mediated human rabies prevention practices. The x-axis shows the effect size (i.e., prevalence), and the standard errors of the effect sizes were plotted on the y-axis. The dashed lines represent the 95% confidence interval. The dots show the distribution of individual studies. Studies with smaller sample sizes are scattered at the bottom of the funnel, and vice versa.

To determine whether there is a possibility of publication bias or small-study effects, we looked at the distribution of studies about the summary effect sizes graphically using funnel plots. Thus, on inspection, the funnel plot showed only a few studies at the bottom, but not a prominent asymmetrical distribution (Figures 6A–C).

However, the formal Egger linear regression test was not statistically significant for a good level of knowledge (β coefficient = −12.17318; standard error = 7.208089; *t* = 0.58; 95% UI: −27.02, 2.67; *p* = 0.571), a favorable level of attitude toward rabies (β coefficient = −5.553584; standard error = 6.659084; *t* = −0.83; 95% UI: −19.30, 8.19; *p* = 0.413), and a good level of rabies prevention practices (β coefficient = 16.41157; standard error = 5.433639; *t* = −0.98; 95% UI: 5.17, 27.65;*p* = 0.357), corroborating the absence of evidence of small study effects.

### Factors associated with KAP towards dog-mediated rabies

From the 27 studies included in the quantitative synthesis, three studies (11.11%) reported factors associated with KAP (29–31). According to a research report by Abdela et al., being male, living in a peri-urban area, attending formal school, having experienced dog bites, owning a dog, and being trained about rabies were associated with higher KAP scores (29). Furthermore, a good level of rabies prevention practices was influenced by a good level of knowledge and attitude toward rabies, having a dog, and getting information from social media, health workers, or training (30, 31) (Table 4).

**Table 4:** Summary of factors associated with good knowledge, favorable attitudes, and good prevention practices toward dog-mediated rabies.

Authors	Outcome/domain	Regression model fitted	Key findings
Ahmed et al. (30)	Knowledge, attitudes, and practices	Multivariable binary logistic regression	Having a good level of knowledge [AOR: 2.41, 95% UI: 2.25, 4.8], a favorable attitudes towards rabies [AOR: 2.06, 95% UI: 1.95, 3.8], owing more than one dog [AOR: 2.46, 95% UI: 1.25, 4.8], living near the veterinary clinic [AOR: 9.32 95% UI: 4.19, 20.70], having access to information from the media [AOR:3.68, 95% UI: 1.74, 7.77], and getting information from health workers [AOR = 3.16, 95% UI:1.60, 6.23] were factors associated with being more likely to report good prevention and control practices.
Wolelaw et al. (31)	Rabies prevention practices	Multivariable binary logistic regression	Being male [AOR: 2.69, 95% UI:1.72, 4.22], being in the 18 to 29 years old age range [AOR: 2.70, 95% UI: 1.20, 6.10], ever bitten by a dog [AOR: 2.40, 95% UI:1.56, 3.68], getting training on rabies [AOR: 1.70, 95% UI: 1.08, 2.68], owning a dog [AOR: 2.92, 95% UI:1.62, 5.26], having good knowledge [AOR: 3.42, 95% UI: 2.19, 5.32], having favorable attitudes towards rabies [AOR: 1.78, 95% UI: 1.16, 2.73], and having a monthly income of more than 2000 Ethiopian birr [AOR: 2.02, 95% UI: 1.28, 3.18] were factors independently associated with good prevention practices of rabies.
Abdela and Teshome (29)	Knowledge, attitudes, and practices	Multivariable binary logistic regression	Being male [AOR: 3.14, 95% UI: 3.01, 9.79], peri-urban residents [AOR: 3.35, 95% UI: 1.98, 11.39], completing secondary school [AOR: 32.8, 95% UI: 2.95, 36.35], experiencing dog bites [AOR: 7.37, 95% UI: 1.83, 29.61], owning a dog [AOR: 7.53, 95% UI: 2.01, 28.19] and being trained about rabies [AOR: 18.62, 95% UI: 1.56, 22.47] were factors independently associated with higher KAP score

AOR, adjusted odds ratio; KAP, knowledge, attitudes and practices; UI, uncertainty interval.

## Discussion

In Ethiopia, dog-mediated rabies has remained a public health challenge, as dog vaccination is neither obligatory nor enforced by law, no policies for controlling stray dogs are in place yet, and most cases of human transmission are due to bites of infected dogs. To our knowledge, this is the first systematic review and meta-analysis mapping evidence on KAP related to dog-transmitted rabies, a well-known NTD, in Ethiopia. The study revealed that the pooled prevalence of a good level of rabies knowledge was estimated to be 62.24%, with individual estimates ranging from 7.50% (39) in the Addis Ababa survey to 99.25% in the Gondar Zuria area (51). The present combined prevalence of a good level of knowledge about rabies is congruent with a recent systematic review in Ethiopia (57), individual study reports in Nigeria (58), and Morocco (59) and is higher than research reports from Thailand (52.1%) (60), Mozambique, Limpopo National Park (18.9%), Bangladesh (58%) (61), and China (56.85%) (62). Such a level of adequate rabies knowledge in Ethiopia is not surprising, given that dog-mediated rabies cases were reported up to an estimated cumulative incidence of 89.8 per 100,000 population in the Tigray region of northern Ethiopia (6) and dog-mediated rabies is endemic in our country. Another justification could be because the majority of studies (67%; *n* = 18 articles) (20, 28, 30, 34, 35, 37, 39, 40, 43–49, 52–54) involved in this meta-analysis were from studies conducted in urban settings, where study participants had better access to health information and communication channels regarding rabies.

The literature points out that negative perceptions about rabies and its control are a major barrier to eliminating dog-mediated rabies mortality (19). This study found that the favorable level of attitudes towards dog-mediated human rabies was only 56.73%, with individual study estimates ranging from 12.55%, in a study conducted around Debretabor town, northern Ethiopia, among community members (53), to 95.57% in a study conducted among dog bite victims in Jimma Health Centre, south-west Ethiopia (49). Our finding was by far lower than studies in Thailand, among primary school children in Chonburi province(89%) (60), and Nigeria, among residents of Abuja Municipal area council, Federal capital territory (74%), however higher than a study by Ossebi et al. (26.3%), among human and animal health professionals, and Herbert et al. (33.5%), among individuals living in urban slums, in India (19). In principle, increasing knowledge will result in changing attitudes and practices to minimize the disease burden (63). However, the negative attitudes reported in our study might reflect the inaccessibility of health facilities with treatment for rabies as well as the cost of the vaccine (64).

Statistical analysis of this study also revealed that the pooled prevalence of a good level of practices related to dog-mediated rabies prevention and control was only 56.73%, with individual study reports ranging from 4.69% (52) in a study from the South Gondar zone, to 85.68% (28) in a study report from Kombolcha, southern Wollo. According to the WHO, vaccinating 70% of dogs in high-risk areas breaks the transmission cycle (10). In this regard, Ethiopia has not taken promising steps forward as evidenced by a dog vaccination rate between 1.8% (53) and 26.9% (65). This can be due to limited access to and availability of dog vaccines (8), the high cost of vaccines (66), poor community participation in pet dog rabies vaccination, and an absence of a veterinary workforce trained in canine mass vaccination strategies or safe dog handling techniques (65). Such sub-optimal dog-mediated rabies prevention and control practices reported in our study are consistent with research results from another canine rabies-endemic country (67). Nevertheless, our finding was far higher than a study from India (68), where only 31.1% would want to apply first aid measures and 36.4% would visit the doctor to avoid the development of rabies. The difference in magnitude of a good level of practices in these settings may be due to study size, sociocultural differences, and the level of engagement of the national policy that fosters zero rabies death global strategy. In the context of Ethiopia, one of the major reasons for the low level of good practice toward dog-mediated rabies prevention and control was the inaccessibility of appropriate post-exposure treatment services (53). In addition, only up to 7.0%–49% (29, 47, 49) applied correct first aid (wash with water and soap) following exposure as an immediate action after a rabid dog bite, and only 4.8% to 21% had ever vaccinated their dog. An estimated 60% of dog victims visit traditional healers (35, 49) for herbal use and water locally called ‘*Tsebel*’ (51). As also pointed out in a recent systematic review by Gelgie et al. (57), we did not find rabies research reports from the Gambela, Benishangul-Gumuz, Afar, and Harai regions or the Dire Dawa city administration. Subgroup meta-analysis based on region revealed the highest level of good knowledge (63%) was reported from the Amhara region, while favorable attitudes (70%) and good prevention practices (70%) were reported from SNNPRs. A lower proportion of good level of knowledge (55%), attitudes (49%), and prevention practices (39%) were reported in Addis Ababa, a city where the cumulative incidence of suspected human rabies exposure cases was estimated at 24.8 cases per 100,000 inhabitants per year (5).

Three studies (25–27) revealed factors associated with KAP among the 27 studies included in this systematic review and meta-analysis. According to Ahmed et al. (30), and Wolelaw et al. (31) a good level of knowledge and a favorable level of attitudes were associated with increased rabies prevention practices. A similar study finding was reported from a rabies-endemic country, Thailand (60, 69), where rabies knowledge and attitudes were strongly and positively associated with rabies preventive practices. This can be justified by the fact that knowledge results in a change in attitudes and practices, which helps decrease disease burden. Higher levels of education and younger age were also associated with a good level of knowledge and favorable attitudes towards rabies (29, 31) which is consistent with a study finding from Morroco (59), and Malawi (70). This could be because educated and younger people tend to have better knowledge and attitudes than illiterate and old people. In addition, male individuals had better KAP scores (29, 31) than females, which is congruent with research from Tanzania (71). This can be justified by the fact that males spend more time outdoors, have better access to schooling than females, and interact with many people, particularly in male supremacist society. Our study also found that regular rabies education (30), and additional training on rabies (29, 31) influence positively rabies prevention practices. In a similar study by Kadowaki et al. (72), supporting these activities, it was suggested that rabies education targeted at minorities/hot spots promoted adequate knowledge, positive attitudes, and good control and prevention practices regarding dog-mediated human rabies. This is justified by the fact that health education and further training on the problem augment or enhance better understandings, perceptions, and behavioral adjustments towards practices.

### Strengths and limitations of the study

This systematic review and meta-analysis findings provide policymakers with an opportunity to examine Ethiopia’s non-reassuring progress towards zero dog-mediated human deaths. Furthermore, our review findings regarding rabies can be used by other countries where rabies is endemic. Methodologically, this study avoided duplication of similar work because the protocol for it was registered, intensive and comprehensive literature searches were conducted to minimize the risk of publication bias, and a double-blinded comprehensive search was conducted over a reasonable period in more than seven online databases to avoid missing published studies. In addition, more than two data abstractors were involved, and to ensure inter-rater agreement, we consulted the Cochrane Handbook for systematic reviews. The newly amended JBI critical appraisal tool was used for quality assessment. Further analyses were conducted to explore sources of dissemination or publication biases. We followed the updated 2020 PRISMA checklist to compile the report. Some limitations have been acknowledged, including the use of different KAP survey tools, and high statistical heterogeneity which requires caution in the interpretation of the findings. In addition, the majority of the participants involved in the primary studies were from urban areas where health information and communication, and access to social and broadcasting services are better. Furthermore, the restriction of language to English needs to be acknowledged.

## Conclusions and recommendations

This systematic review and meta-analysis provide robust information from available data, regarding knowledge, attitudes, and prevention practices towards dog-mediated human rabies in Ethiopia. The REM revealed that there were low levels of favorable attitudes and of good practices about the problem. Furthermore, the analysis found some level of good knowledge was exhibited among community members and dog bite victims. Best practices in dog-mediated human rabies control require the establishment of rabies post-exposure treatment guidelines and their dissemination to all tiers of the health care delivery system and a sound monitoring and supervision system. Therefore, inter-sectoral collaboration among healthcare workers, policymakers, researchers in the areas of NTD, community leaders, veterinary professionals, and environmental professionals should be put in place to contribute to the sustainable development goal of achieving zero human deaths from dog-mediated rabies by 2030.

## Data availability statement

The original contributions presented in the study are included in the article/Supplementary material, further inquiries can be directed to the corresponding author.

## Author contributions

BW: Conceptualization, Data curation, Formal analysis, Investigation, Methodology, Project administration, Resources, Software, Supervision, Validation, Visualization, Writing – original draft, Writing – review & editing. APG: Conceptualization, Data curation, Formal analysis, Investigation, Methodology, Project administration, Resources, Software, Supervision, Validation, Visualization, Writing – review & editing. AYG: Conceptualization, Data curation, Formal analysis, Investigation, Methodology, Project administration, Resources, Software, Supervision, Validation, Visualization, Writing – review & editing. GK: Conceptualization, Data curation, Formal analysis, Investigation, Methodology, Project administration, Resources, Software, Supervision, Validation, Visualization, Writing – review & editing. GA: Conceptualization, Data curation, Formal analysis, Investigation, Methodology, Project administration, Resources, Software, Supervision, Validation, Visualization, Writing – review & editing. YA: Formal analysis, Investigation, Methodology, Project administration, Resources, Software, Supervision, Validation, Visualization, Writing – review & editing, Conceptualization, Data curation.

## Footnotes

^1^ https://www.crd.york.ac.uk/prospero/display_record.php?ID=CRD42023437439
