# Supplementary material for: Knowledge, attitudes and prevention practices related to dog-mediated rabies in Ethiopia: a systematic review and meta-analysis of observational epidemiological studies from inception to 2023
Source: Front Public Health. 2023 Dec 21;11:1276859. doi: 10.3389/fpubh.2023.1276859 (PMC10764596; doi:10.3389/fpubh.2023.1276859)
Supplement: Supplementary file 1 [file Table_1.docx]

**S1Table: Search detail and concept map**

| **Concept map 1:**Knowledge | Keywords | Knowledge [Text Word] |
| --- | --- | --- |
|  | MeSH terms | "Knowledge"[Mesh] |
| **Concept map 2:** Attitude | Keywords | "Attitude"[text word] |
|  | MeSH terms | "Attitude"[Mesh] |
| **Concept map 3:**Practice | Keywords | Practice [Text Word] |
|  | MeSH terms | "Health Knowledge, Attitudes, Practice"[Mesh] |
| Concept map 4: Rabies | Keywords | rabies[Text Word] |
|  | MeSH terms | "rabies"[MeSH Terms] |

("knowledge"[MeSH Terms] OR "knowledge"[All Fields] OR "knowledge s"[All Fields] OR "knowledgeability"[All Fields] OR "knowledgeable"[All Fields] OR "knowledgeably"[All Fields] OR "knowledges"[All Fields]) AND ("attitude"[MeSH Terms] OR "attitude"[All Fields] OR "attitudes"[All Fields] OR "attitude s"[All Fields]) AND ("practicability"[All Fields] OR "practicable"[All Fields] OR "practical"[All Fields] OR "practicalities"[All Fields] OR "practicality"[All Fields] OR "practically"[All Fields] OR "practicals"[All Fields] OR "practice"[All Fields] OR "practice s"[All Fields] OR "practiced"[All Fields] OR "practices"[All Fields] OR "practicing"[All Fields]) AND ("rabies"[MeSH Terms] OR "rabies"[All Fields])

Or

(((knowledge) AND (attitude)) AND (practices)) AND (rabies)
