# Supplementary material for: Knowledge, attitudes and prevention practices related to dog-mediated rabies in Ethiopia: a systematic review and meta-analysis of observational epidemiological studies from inception to 2023
Source: Front Public Health. 2023 Dec 21;11:1276859. doi: 10.3389/fpubh.2023.1276859 (PMC10764596; doi:10.3389/fpubh.2023.1276859)
Supplement: Supplementary file 3 [file Table_3.docx]

**S3 Table** : Quality assessment of studies using JBI’s critical appraisal tools designed for Descriptive cross-sectional study

| Study | Sample size | JBI’s critical appraisal questions | | | | | | | | | | | | | | | | | Score | Overall Appraisal |
| --- | --- | --- | --- | --- | --- | --- | --- | --- | --- | --- | --- | --- | --- | --- | --- | --- | --- | --- | --- | --- |
|  |  | Q1 | Q2 | | Q3 | | Q4 | | Q5 | | Q6 | | Q7 | | Q8 | | Q9 | |  |  |
| Alie et al | 384 | Y | | Y | | Y | | Y | | U | | Y | | Y | | Y | | Y | 8 | Included |
| Hagos et al | 633 | Y | | Y | | Y | | Y | | Y | | Y | | Y | | U | | Y | 8 | Included |
| Guadu et al | 410 | Y | | Y | | Y | | Y | | Y | | Y | | Y | | Y | | Y | 8 | Included |
| Yalemebrat et al | 416 | Y | | Y | | Y | | Y | | Y | | Y | | Y | | N | | Y | 8 | Included |
| Gebremeskel et al | 384 | Y | | Y | | Y | | Y | | Y | | Y | | Y | | Y | | Y | 9 | Included |
| Jemberu et al | 120 | Y | | Y | | Y | | Y | | Y | | Y | | Y | | Y | | Y | 9 | Included |
| Bahiru et al | 889 | Y | | Y | | Y | | N | | Y | | Y | | Y | | Y | | Y | 8 | Included |
| Kabeta et al | 384 | Y | | Y | | Y | | Y | | y | | N | | Y | | Y | | Y | 8 | Included |
| Abdela et al | 135 | Y | | Y | | Y | | Y | | Y | | N | | Y | | N | | Y | 8 | Included |
| Digafe et al | 400 | Y | | Y | | Y | | Y | | Y | | Y | | Y | | Y | | N | 8 | Included |
| Bihon et al | 384 | Y | | Y | | Y | | Y | | Y | | Y | | Y | | N | | Y | 8 | Included |
| Ali et al | 1240 | Y | | Y | | Y | | Y | | Y | | Y | | Y | | N | | Y | 8 | Included |
| Mamuye et al | 410 | Y | | Y | | Y | | Y | | Y | | Y | | Y | | N | | Y | 8 | Included |
| Gebeyaw et al | 138 | Y | | Y | | Y | | Y | | Y | | Y | | Y | | N | | Y | 8 | Included |
| Newayeselassie et | 315 | Y | | Y | | Y | | Y | | Y | | Y | | Y | | N | | Y | 8 | Included |
| Tolosa and Mengistu | 384 | Y | | Y | | Y | | Y | | Y | | Y | | Y | | N | | Y | 8 | Included |
| Birasa et al | 400 | Y | | Y | | Y | | Y | | Y | | Y | | Y | | N | | Y | 8 | Included |
| Gumi et al | 162 | Y | | Y | | Y | | Y | | Y | | Y | | Y | | N | | Y | 8 | Included |
| Abera et al | 384 | Y | | Y | | Y | | Y | | Y | | Y | | Y | | N | | Y | 8 | Included |
| Dabuma et al | 386 | Y | | Y | | Y | | Y | | Y | | Y | | Y | | N | | Y | 8 | Included |
| Jama and mengistu | 384 | Y | | Y | | Y | | Y | | Y | | Y | | Y | | N | | Y | 8 | Included |
| Fesseha and Abebe | 330 | Y | | Y | | Y | | Y | | Y | | Y | | Y | | N | | Y | 8 | Included |
| Tamiru et al | 633 | Y | | Y | | Y | | Y | | Y | | Y | | Y | | N | | Y | 8 | Included |
| Wassihune et al | 360 | Y | | Y | | Y | | Y | | Y | | Y | | Y | | N | | Y | 8 | Included |

Y –Yes;N-No;U -Unclear-Question. Overall score is calculated by counting the number of Y’s in each row.Q1=Was the sample frame appropriate to address the target population? Q2=Were study participants sampled in an appropriate way? Q3=Was the sample size adequate? Q4=Were the study subjects and the setting described in detail? Q5=Was the data analysis conducted with sufficient coverage of the identified sample? Q6=Were valid methods used for the identification of the condition? Q7=Was the condition measured in a standard, reliable way for all participants? Q8=Was there appropriate statistical analysis? Q9=Was the response rate adequate, and if not, was the low response rate managed appropriately?
