# Supplementary material for: Knowledge, attitudes and prevention practices related to dog-mediated rabies in Ethiopia: a systematic review and meta-analysis of observational epidemiological studies from inception to 2023
Source: Front Public Health. 2023 Dec 21;11:1276859. doi: 10.3389/fpubh.2023.1276859 (PMC10764596; doi:10.3389/fpubh.2023.1276859)
Supplement: Supplementary file 4 [file Table_4.docx]

**S4 Table**: Quality assessment of studies using JBI’s critical appraisal tools designed for Analytical cross-sectional study

| Study | Sample size | JBI’s critical appraisal questions | | | | | | | | Score | Overall Appraisal |
| --- | --- | --- | --- | --- | --- | --- | --- | --- | --- | --- | --- |
|  |  | Q1 | Q2 | Q3 | Q4 | Q5 | Q6 | Q7 | Q8 |  |  |
| Ahmed et al | 326 | Y | y | y | y | y | y | y | y | 8 | Included |
| Wolelaw et al | 609 | y | y | y | y | y | y | y | y | 8 | Included |
| Abdela and Teshome | 150 | Y | y | y | y | y | y | y | y | 8 | Included |

Y –Yes;N-No;U -Unclear-Question. Overall score is calculated by counting the number of Y’s in

For analytical cross-sectional study, the JBI checklist assessed the following questions

Major components :

1. Were the criteria for inclusion in the sample clearly defined?

2. Were the study subjects and the setting described in detail?

3. Was the exposure measured in a valid and reliable way?

4. Were objective, standard criteria used for measurement of the condition?

5. Were confounding factors identified?

6. Were strategies to deal with confounding factors stated?

7. Were the outcomes measured in a valid and reliable way?

8. Was appropriate statistical analysis used?
